# Tri-Band Rectenna Dedicated to UHF RFID, GSM-1800 and UMTS-2100 Frequency Bands

**DOI:** 10.3390/s22093565

**Published:** 2022-05-07

**Authors:** Ognadon Assogba, Abdoul Karim Mbodji, Arnaud Bréard, Abdou Karim Diallo, Yvan Duroc

**Affiliations:** 1 Section Physique Appliquée, UFR Sciences et Technologies, Université Gaston Berger de Saint-Louis, Saint-Louis BP 234, Senegal; assogba.ognadon@ugb.edu.sn (O.A.); abdoul-karim.mbodji@ugb.edu.sn (A.K.M.); abdou-karim.diallo@ugb.edu.sn (A.K.D.); 2 UMR5005, Ampère, CNRS, Université Claude Bernard Lyon 1, INSA Lyon, Ecole Centrale de Lyon, Univ Lyon, 69130 Ecully, France; arnaud.breard@ec-lyon.fr

**Keywords:** rectenna, antenna, rectifier, energy harvesting, radio frequency

## Abstract

The omnipresence of connected objects leads to the quasi-permanent presence of electromagnetic waves from different sources in our environment. This article presents a new electromagnetic energy harvesting device, rectenna type, which offers the advantage of being versatile. Indeed, the proposed prototype is compatible with three frequency bands of radio standards widely deployed today (UHF RFID, GSM-1800, and UMTS-2100), and its performances remain good for low to very low ambient power levels as well as for different loads depending on the targeted application. The proposed solution is based on a tri-band antenna with very good efficiency and a bandwidth of at least 80 MHz for each of the operating frequencies. Moreover, the associated rectifier circuit is also tri-band and offers good performance in terms of RF-to-DC conversion efficiency for input levels varying in a rather wide range of power levels. The study is based on a design phase by simulation until the realization of prototypes and their experimental characterization. The designed rectenna is compared with solutions found in the literature.

## 1. Introduction

With the development of the Internet of Things and the ubiquity of connected objects, the energy autonomy of wireless sensors is a major issue, the importance of which will continue to grow in the years to come. The traditional use of an on-board energy source (such as a rechargeable battery or cell) implies larger and heavier devices, which also limits their lifespan or require maintenance. What’s worse, batteries are already extremely widespread and difficult to recycle; the multiplication of their use would constitute an ecological threat and would be contrary to the desire for the sustainable development of wireless technologies.

Among the possible alternatives [1,2,3,4,5], the recovery of electromagnetic energy appears to be a relevant solution because it exploits the radio frequency (RF) waves of telecommunications already present in the environment. It is also a way to improve the efficiency of use of electromagnetic waves emitted for other applications, and thus to avoid a certain waste. The rectenna, which is an antenna associated with an RF-to-DC converter circuit (i.e., a rectifying antenna), is the basic element for collecting electromagnetic energy. This concept, invented in the 1960s, is more than ever the subject of numerous works [6].

**Figure 1:**
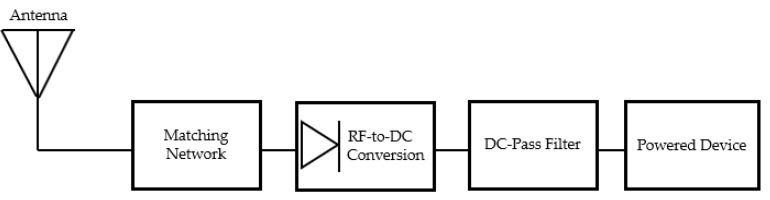
Typical block diagram of a RF energy harvesting system.

It is a question, on the one hand, of “capturing” one or different frequencies coming from radio technologies likely to be present in the targeted application environment and, on the other hand, of increasing the conversion efficiency; and thus, of being able to supply the quantity of energy necessary to power a sensor or any other device (e.g., an actuator). To do this, the RF energy recovery systems are mainly made up of a receiving antenna in charge of picking up one or more frequencies, an impedance matched rectifier in charge of converting RF into DC, and a DC pass filter in charge of filtering harmonics, as shown in Figure 1.

With the objective of being able to provide the energy and/or voltage level required to power the device, from this general functional configuration, several options are possible and many solutions have been proposed in the literature. In practice, the wireless radio technologies assumed to be present in the application environment and the energy requirements of the device to be powered will guide the design and specify the constraints: mainly, the type of antennas (i.e., single band, multi-band, or broadband antenna) and the rectifier architecture (e.g., series, doubler, Greinacher, or Graetz architecture) [7,8,9,10]. Note that using series structures results in high conversion efficiency and low output voltage, while using multiple rectifier diodes decreases conversion efficiency but results in increased load terminal voltage. Schottky diodes are most commonly used for RF-to-DC conversion because of their low forward threshold voltage and very short switching time, which allows for the detection of low power RF signals. Nowadays, diode-connected MOS transistors are widely used as signal rectifiers. The matching network allows the impedance matching between the antenna and the rectifier in order to optimize the power transfer. This circuit can be either lumped or distributed elements, or even a combination of lumped and distributed elements. The use of distributed elements such as microstrip lines avoids the soldering and parasitic losses that are observed in lumped elements, but the circuit size is increased.

For illustration, in [10], a rectangular antenna consisting of a patch antenna operating in the 2.45 GHz frequency band with good performance is presented. The antenna has a bandwidth of 850 MHz, a gain of 2.4 dB, and a radiation efficiency of 98.93%. The dual-voltage rectifier and associated matching circuit with lumped and distributed elements achieve a maximum conversion efficiency of 62% at an input power of 0 dBm. Since RF services are not always deployed everywhere and the RF power levels recovered from the environment are also in the milli-watt range, the current trend is to design multi-band rectennas taking into account the RF technologies present in the application environment, e.g., UHF RFID, mobile telephony (3G, 4G, …), GSM-900, GSM-1800, UMTS-2100, CDMA, or Wi-Fi bands [11,12,13,14,15,16]. It is worth noting that the 900 and 1800 bands present a dominant spectrum with high power densities compared to Digital TV, UMTS, and Wi-Fi bands [16,17].

The antenna remains generally a single element integrating the different frequency bands of interest. However, the rectification part (including matching and rectification) allows several approaches. For instance, for a better RF-to-DC conversion efficiency at the expense of complexity and size, for each of the frequencies of interest, a matching circuit and a rectifying circuit are designed and associated [18,19]. To simplify the architecture, with the constraint of keeping good conversion performances, it is also possible to design either a single matching circuit for the multiple rectifiers [20], a single rectifying circuit preceded by several matching circuits, or (ideal case) a single matching circuit associated with a single rectifying circuit [21,22,23].

This study proposes a new, low-cost tri-band rectenna operating in UHF RFID, GSM-1800 and UMTS-2100 frequency bands. The waves operating in these three frequencies bands come from RFID applications and access networks of telephone operators deployed in the human environment. If at 900 MHz, RFID technology is more particularly targeted because it has become one of the standards for the Internet of Things and wireless sensor networks and because of the authorized emission levels (up to 36 dBm), cellular telephony, which is widely deployed, is also a possible RF source. These waves are therefore permanent in the ambient environment and so are potential candidates for sources of RF energy harvesting. The proposed solution aims at obtaining an efficient energy harvesting device comprising a multi-band monopole antenna, a distributed multi-band matching circuit and a single rectifier circuit based on a single serial voltage topology. Section 2 presents the complete design of the rectenna by distinguishing the radiative part (i.e., the antenna) and the rectifying part (i.e., rectifier and matching circuit). The different steps of the design of the multi-band antenna are detailed to underline the method followed, which allows in particular to obtain a compact antenna. The topology of the rectification part is also detailed. Section 3 shows and compares the results obtained in simulation and experimentally. In particular, the reflection coefficient of the antenna and its radiation pattern are illustrated, as well as the conversion efficiency of the rectifier for different loads and over a wide range of input power levels of the rectifier. Section 4 discusses the overall performance of the proposed rectenna based on a detailed comparison with other rectennas from the literature. Finally, Section 5 presents the conclusion and perspectives of this study.

## 2. Tri-Band Rectenna Design

The objective is to design a low-cost tri-band rectenna both operating in UHF RFID, GSM-1800 and UMTS-2100 frequency bands. The design of the rectenna is divided into two main steps: the design of the antenna (Section 2.1), whose role is to capture the electromagnetic waves in the bands of interest; the design of the rectifier circuit associated with the matching circuit (Section 2.2), whose role is the conversion of the captured RF waves into DC power for a given load, e.g., a sensor.

Note that the high frequency simulation software (HFSS-version 13.0) and the Advanced Design System (ADS-version 2017) are used for the antenna design and the rectifier design respectively.

### 2.1. Antenna Design Steps

In order to obtain an easy to manufacture and low-cost antenna, the antenna structure is chosen to be a printed monopole antenna with a rectangular shape. The radiating element and the 50 Ω microstrip supply line are simply etched on the substrate, as well as the ground plane on the opposite side. For this study, the substrate is an FR4 type substrate with a thickness, h, of 1.6 mm, a relative permittivity, ${Ɛ}_{r}$, of 4.4, and a loss tangent of 0.02. The thickness of the copper is 35 µm.

To design the printed monopole, the first antenna structure designed is a rectangular patch antenna. One of the advantages for this type of antenna is that there are theoretical equations for determining the main dimensions [10]. According to these theoretical equations, the dimensions of the radiating element (W × L) are 101.40 mm × 79.22 mm. For the substrate, the initial dimensions were arbitrarily chosen equal to 111 mm × 118 mm.

In order to reduce the size of the antenna, a series of simulations was performed with the criteria of obtaining a radiation efficiency higher than 80% and a reflection coefficient lower than 10 dB. The variable parameters were the dimensions of the radiating element, the substrate, and the feed line. For reasons of simplicity, the feed line is a microstrip line placed on the same plane as the radiating element: the notches help to realize the impedance matching and as inspired from [24] the feed line is not centered. Finally, the optimized dimensions of the radiator which is asymmetric [25] and substrate were determined to be equal to 42 mm × 37.2 mm and 78 mm × 50 mm respectively.

**Figure 2:**
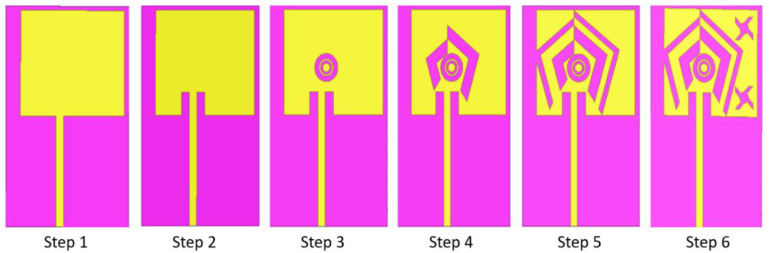
Antenna design steps: successive modification of the antenna geometry until the proposed solution.

Figure 2 shows the different steps of the antenna design, from step 1 to step 6. It should be noted that each design step has a significant influence on the antenna parameters, especially on the impedance matching and the radiation efficiency, because the modification of the antenna leads to a new surface current distribution on the radiating element. Consequently, the performance is evaluated for each step and allows to guide the optimization. In the design of the antenna from step 1 to step 4, the ground plane had a single ground plane element of width 9 mm. This ground plane has been modified from step 5 onwards by adding a second element 9 mm away from the first one. The idea behind the creation of the two ground plane elements was to create a new current distribution (and thus the appearance of a new frequency band) with the modification of the radiator. Step 1 corresponds to the initial optimized configuration with a non-centered feed line. The microstrip feeding line has been used for excitation of the antenna. To improve the matching of the antenna with the feed line, two slots were created in step 2. We then created concentric ring discontinuities on the radiator in step 3. Two pairs of rectangular V-shaped slots are added from step 4 to step 5. Step 6 shows the final optimized antenna configuration for the three resonant frequencies. The V-shapes were extracted from the sides of the concentric pentagons. The objective in choosing the V-shaped slots is to obtain a current intensification at the corners of these discontinuities. The purpose of creating slots in steps 3 and 4 is to promote an intensification of the areal current distribution on the radiating element. The slots in steps 5 and 6 are intended to introduce a new current distribution on the radiating element for the desired third resonant frequency.

**Figure 3:**
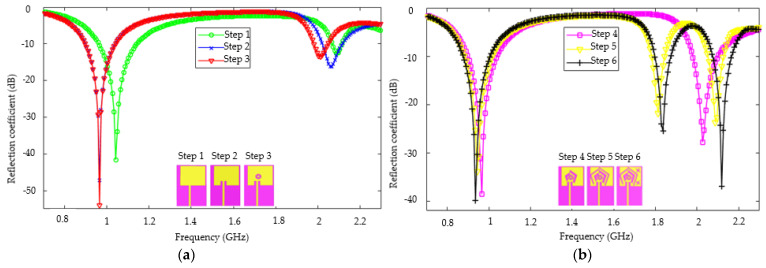
Reflection coefficient of the proposed antenna versus frequency: (**a**) from step 1 to step 3 and (**b**) from step 4 to step (6).

Figure 3 shows the variations of the reflection coefficient versus the frequency for each step of the antenna design. From step 1 to step 4, the antenna operates in two frequency bands. In step 2, the reflection coefficient of the antenna has been improved compared to the first step around 0.9 GHz. Indeed, the creation of two symmetrical slots with respect to the feed line would have led to an improvement of the monopole antenna matching with the feed line. The creation of ring slots in step 3 and two pairs of V-shaped slots in step 4 has improved the antenna matching around 0.9 GHz. The third frequency band of 1.8 GHz appeared from step 5 to step 6. The appearance of this frequency can be explained by the pair of discontinuities created in step 5. Indeed, the creation of V-shaped slots led to the appearance of a significant surface current distribution at certain points of these slots.

**Figure 4:**
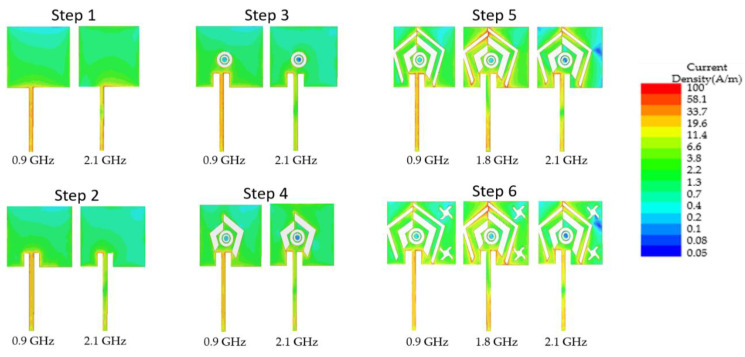
Current distribution on the radiator and the feedline from step 1 to step 6.

Figure 4 shows the surface current distribution on the radiator for resonance frequencies with a reflection coefficient lower than −10dB. This current distribution leads to the impedance matching of the antenna at 1.8 GHz. Based on the modulus of the surface current distribution (A/m^2^), one conclusion is that the appearance of a current distribution with a high current density at a point of the radiating element can induce an impedance matching of the antenna and the appearance of a resonant frequency. The new structure of the ground plane would have had as effect, the appearance of a new distribution of charges on the radiator (repulsive effects), which would have entrained a new distribution of the current on the radiator. Step 6 shows a better antenna matching and an increase of the antenna bandwidth at the three frequency bands 0.9, 1.8, and 2.1 GHz. The presence of a fairly large current distribution at the 0.9 GHz frequency band should also be noted in Figure 4.

**Figure 5:**
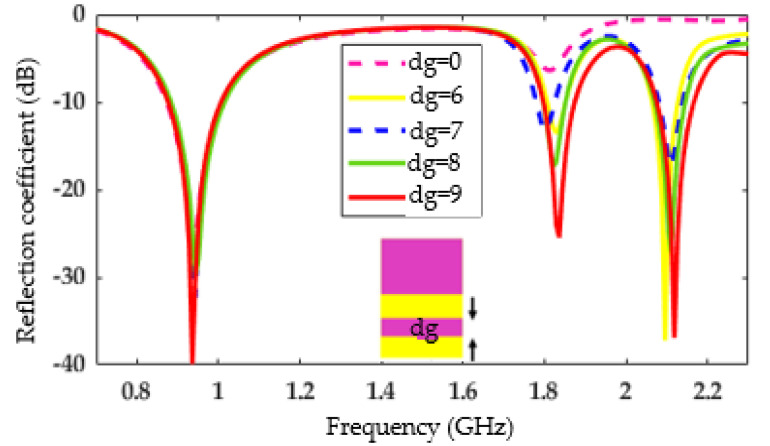
Influence of the parameter, dg, on the reflection coefficient.

Furthermore, an additional antenna design step concerns the other side of the radiating element, specifically the ground plane. The original ground plane proposed here is divided into two parts separated by a distance dg, as illustrated in Figure 5 (and more clearly in Figure 8a). This separation distance has a significant influence on the reflection coefficient. Figure 5 shows the reflection coefficient versus frequency for variations of this distance: dg varies from 0 mm (the two parts of the ground plane are then joined) to 9 mm (value found as optimal). Note that beyond a distance greater than 9 mm, a degradation of the reflection parameters is observed.

Table 1 specifies, for design steps 5 and 6 (for which all three operating bands are present), the resonant frequency, reflection coefficient value, bandwidth, and efficiency for each of the bands. The integration of star slots in step 6 significantly increased the antenna performance, such as the reflection coefficient and radiation efficiency. Thus, the integration of slots on the antenna leads not only to the improvement of the antenna parameters, but also to the appearance of frequency bands when there is a significant density current distribution at a point on the radiating element. The designed antenna thus operates at resonant frequencies of precisely 0.93 GHz, 1.83 GHz, and 2.11 GHz with, respectively, a maximum realized gain of 5.99 dB obtained at the frequency of 2.11 GHz and a maximum radiation efficiency of 98.49%.

**Figure 6:**
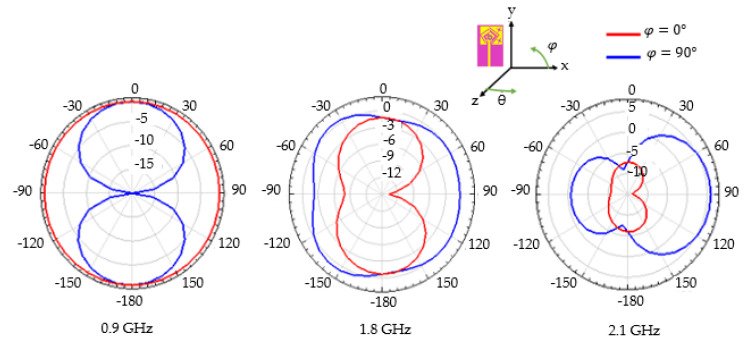
Simulated 2D radiation pattern of the far-field tri-band antenna gain for the three operating frequencies: 0.9, 1.8 and 2.1 GHz.

Figure 6 shows the simulated radiation pattern of the far-field tri-band antenna gain for the E-plane (φ=0°) and the H-plane (φ=90°). The printed monopole antenna behaves differently according to its frequency band and according to its plane. For the resonant frequency equal to 0.9 GHz, the observed pattern is similar to that of a dipole antenna. For the frequency 1.8 GHz, the pattern is quasi-omnidirectional in the H plane, while at the frequency 2.1 GHz it is directional. The gain of the antenna is equal to −0.6, 0.8, and 5.86 dB respectively for the frequencies of 0.9, 1.8, and 2.1 GHz. Note that these gain values for the first two frequency bands are low and were not expected with the obtained radiation efficiency values. Although the measured values (presented in Section 3) of the gains are more favorable, they corroborate the simulations. Thus, it appears that the efficiency is overestimated by the HFSS software, the electromagnetic software used for this study. However, this simulation design leads to a compact antenna with three resonance bands: the first two bands (at 0.9 and 1.8 GHz) have an almost omnidirectional radiation pattern while the highest band (at 2.1 GHz) has a more directional pattern and high gain.

### 2.2. Rectifier Design

**Figure 7:**
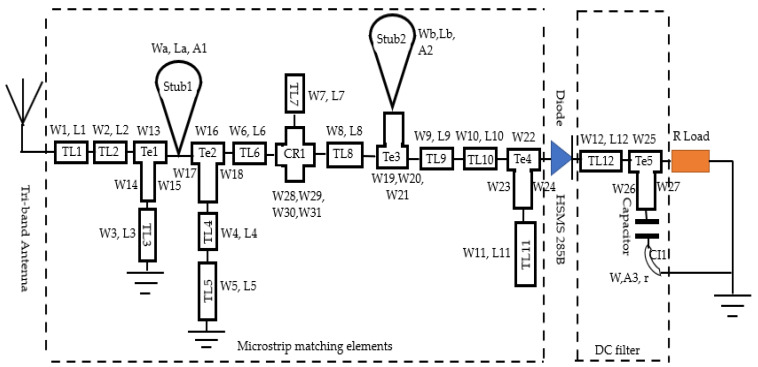
Topology of triple band rectifier.

Figure 7 shows the designed rectifier circuit associated with its impedance matching circuit, which is a distributed circuit. Table 2 details the values of the dimensions of each microstrip line element: the lengths (W, L, r) are given in mm and the angles (A) in degrees.

For this work, the rectifier is a simple circuit composed of a rectifier diode which is associated with a capacitor in parallel with a load resistor. The rectifier diode here used is the Schottky diode HSMS 285B whose characteristics are: threshold voltage 0.25 V, series resistance 25 Ω, and minimal junction capacitance 0.18 pF. The rectifier capacitor has been set with a capacitance value of 100 pF while the load resistance, Rload, is assumed to be variable. The source associated with the rectification circuit being the tri-band antenna, the following impedance values (Z(f)) for the three resonant frequencies are considered:

Z(0.9 GHz)=46.86−1.89j Ω;

Z(1.8 GHz)=45.1−1.56j Ω;

Z(2.1 GHz)=46.1+3.97j Ω

These impedances are given at these frequencies of interest in the HFSS software.

The impedance matching at the rectifier level is obtained by using the Smith chart matching component between the antenna and the rectifier and the Smith chart tool to determine the line elements ensuring a better impedance matching. Then, the *linecalc* tool is used to determine the dimensions of each line element composing the matching circuit. In addition, in order to remove some harmonics and ensure the operation of the rectifier in the three bands, a filter made of line elements has also been added. All these matching, rectifying, and filtering elements are etched on the FR4 type substrate (thickness, h, of 1.6 mm, relative permittivity, ${Ɛ}_{r}$, of 4.4, and loss tangent of 0.02). The thickness of the microstrip lines and ground plane is 35 µm.

It is also worth noting that the software ADS integrates the nonlinear circuit simulation tool named harmonic balance (HB) simulator which is used to integrate S-parameters of the antenna [26].

## 3. Experimental Evaluation of the Proposed Tri-Band Rectenna

### 3.1. Performance of the Fabricated Tri-Band Antenna

**Figure 8:**
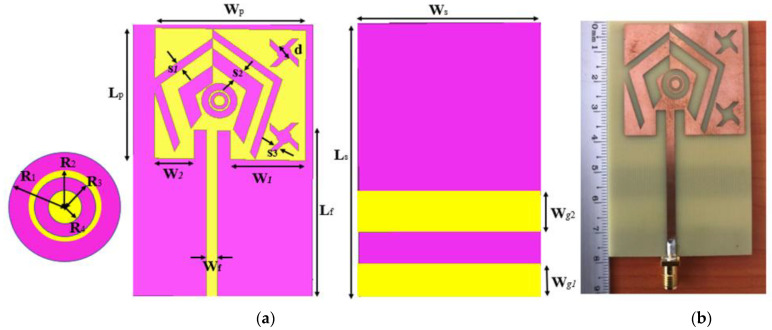
Proposed tri-band antenna: (**a**) optimized design; (**b**) photo of the fabricated prototype.

Figure 8a shows the detailed geometry of the optimized antenna and Figure 8b shows a photo of the fabricated tri-band antenna. Table 3 gives the values of the associated different parameters. It should be also noted that a distance of 0.2 mm between the V-slots of the same pair is ensured in order to solve the potential occurrence of open circuits due to inaccuracies in the manufacturing process.

**Figure 9:**
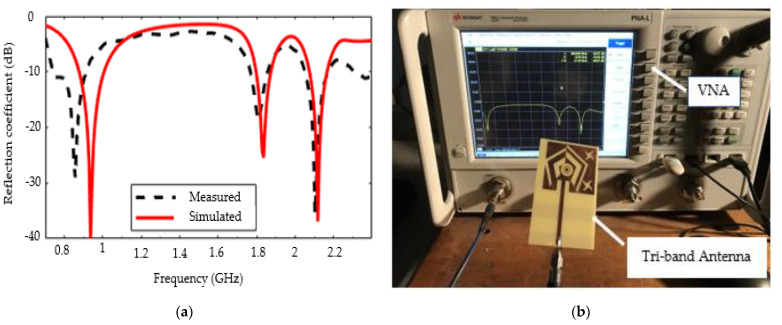
Reflection coefficient: (**a**) Simulated and measured results (in dB); (**b**) experimental setup.

Figure 9a shows the simulated and measured reflection coefficient of the monopole as a function of frequency. Note that measurements were made using a vector network analyzer (VNA) and in an anechoic chamber (not visible on the photo of Figure 9b). The measurement results show that the realized monopole works at resonant frequencies of 0.85 GHz, 1.8 GHz, and 2.1 GHz with a reflection coefficient of −28.95, −18.52, and −35.58 dB, respectively. For the two highest frequencies, the results show good agreement between simulation and measurement. There is a slight shift of the 74 MHz resonant frequency towards low frequency with an increase of the reflection coefficient compared to the simulation around 0.9 GHz, and more favorably, a slight increase of 6.5% in bandwidth. This frequency shift is probably due to the soldering of the SMA connector used during the manufacturing process. Indeed, the microstrip line on which a branch of the connector is fixed also radiates, and in particular, around 0.9 GHz, a strong surface current distribution is observed on the line. The slight decrease in the observed reflection coefficient can also be explained by the losses in the cables and in the SMA connector used.

**Figure 10:**
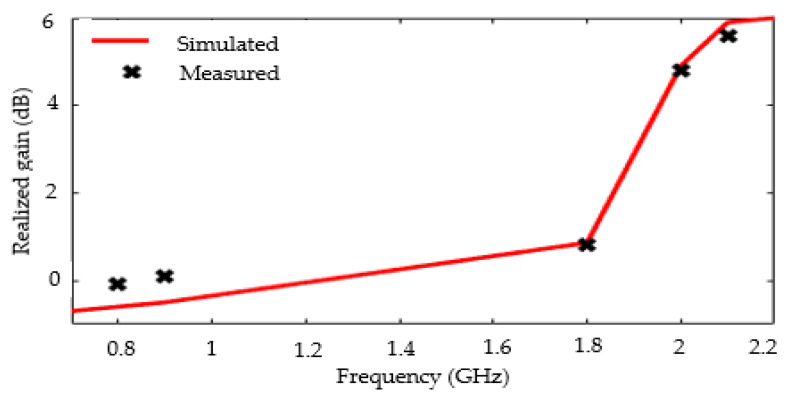
Simulated and measured realized gain of the tri-band antenna as function of frequency.

Finally, Figure 10 shows the simulated and measured realized gain of the tri-band antenna as a function of frequency. The measurement results are therefore in agreement with those of the simulation. The measured realized gain is 0.2, 0.8, and 5.6 dB at 0.9, 1.8, and 2.1 GHz, respectively.

### 3.2. Performance of the Fabricated Rectifier

**Figure 11:**
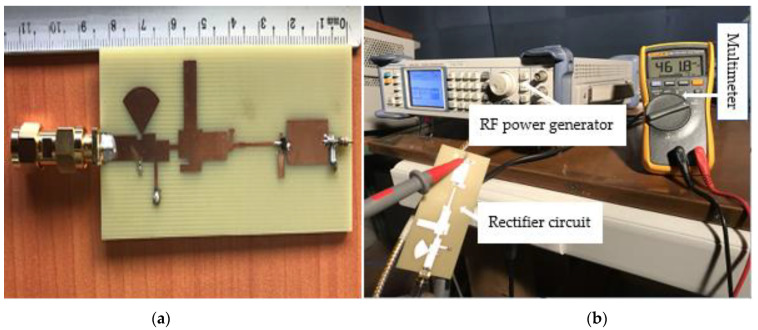
RF-to-DC conversion: (**a**) photo of the prototype of the manufactured rectifier; (**b**) experimental setup for the performance evaluation.

Figure 11a shows a photo of the rectifying circuit (including the impedance matching part) manufactured with a SMA connector for test and performance evaluation. Figure 11b illustrates the used experimental setup.

In order to study the impact of the load resistance and determine its optimal value, the RF-to-DC conversion efficiency, for each of the three frequency bands, as a function of the load resistance is determined from measurement for an input power set to −5 dBm. The conversion efficiency, η, at the rectifier terminals is obtained by Equation (1):

(1) $$\eta \left(\%\right)=\frac{V_{out}{}^{2}}{P_{in}\times R_{load}}\times 100$$

where $V_{out}$ (in Volt) is the voltage on the resistor, $R_{load}$ is the value of the resistor, and $P_{in}\;$ the input power (in Watt) of the receiving antenna.

**Figure 12:**
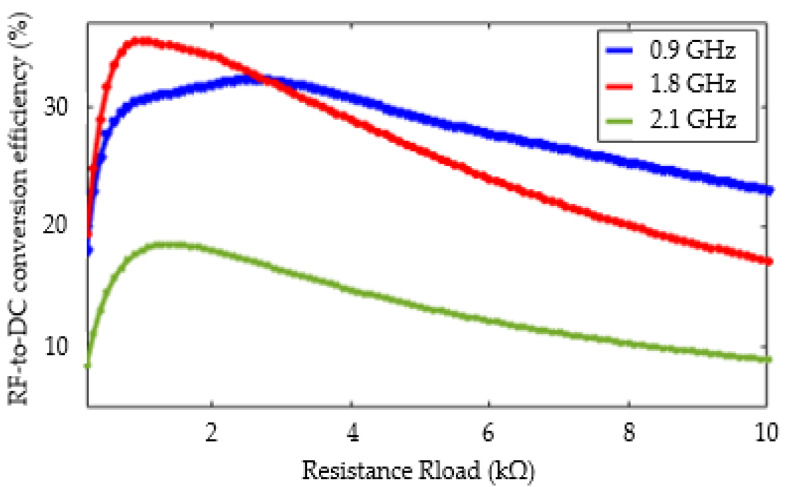
RF-to-DC efficiency simulation as function of the resistance load for an input power set to 0 dBm.

Figure 12 shows the obtained results. The efficiency curves for the three operating frequencies have a similar behavior: when the value of the load impedance increases, they increase to a maximum and then decrease. The optimal values of the RF-to-DC conversion efficiency are 32.3%, 35.5%, and 18.6%, respectively for 0.9, 1.8, and 2.1 GHz. The load resistance corresponding to the optimal values of the conversion efficiency is 2.6, 1, and 1.4 kΩ respectively at frequencies of 0.9, 1.8, and 2.1 GHz. GHz. It is interesting to note that for the 0.9 GHz frequency, a good robustness with respect to the load variation is obtained. The efficiency remains above 30% when the impedance varies from 0.3 to 4 kΩ. For the rest of the study, the load impedance is chosen equal to 1.8 kΩ, a value that leads to a good compromise for the three resonance frequencies.

**Figure 13:**
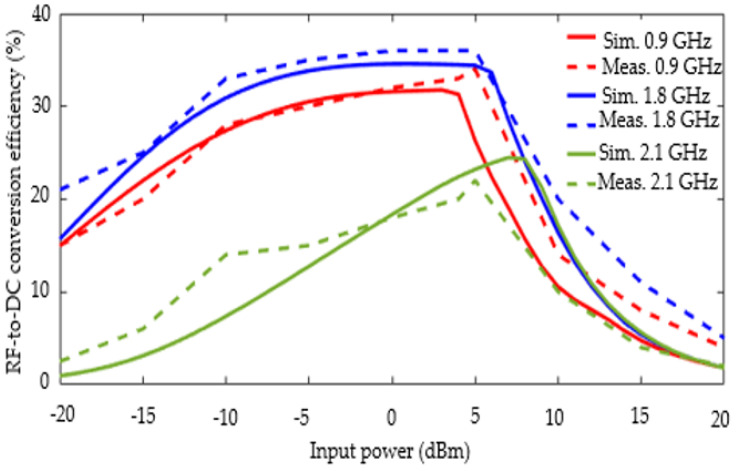
Simulated and measured RF-to-DC efficiency as function of the input power when the load resistance is equal to 1.8 kΩ.

Considering this load impedance, Figure 13 shows the simulated and measured RF-to-DC conversion efficiency as function of the input power varying between −20 to 20 dBm. The simulation and measurement results are in good agreement. Note that the analysis below is based on the measurement results. As expected from the previous result, the maximum RF-DC efficiency conversion (which is equal to 35%) is obtained at the 1.8 GHz frequency. For this frequency, the efficiency is almost constant and higher than 33% for an input power ranging from −10 dBm to +5 dBm. On this same power range, for the frequency of 0.9 GHz, the efficiency is also almost constant and higher than 26% while at 2.1 GHz, the efficiency varies almost linearly from about 13% to 20%. In all three cases, from 5 dBm, the efficiency decreases quite strongly. In addition, it should also be noted that, for the two low frequency bands, the RF-DC conversion efficiency remains quite high at low input powers. It should be noted that this constitutes one of the most important advantages of the proposed solution. For example, when the input power is only equal to −20 dBm, the efficiency is 15% and 21% for 0.9 GHz and 1.8 GHz, respectively.

## 4. Synthesis of the Characteristics of the Proposed Tri-Band Rectenna Compared with the Literature

Table 4 and Table 5 present a comparative study with previous works in the literature. For clarity, the characteristics of both the antenna and the rectifier are presented. Table 4 presents the performance of the antenna in terms of dimensions, frequency bands (value of the center frequency and number of bands), gain for each band, and efficiency. Table 5 compares the rectifier circuits by showing the frequency bands, the number of ports (translating whether the circuit operates for all bands or whether a specific circuit addresses a particular band), the structure of the rectifier circuit and the type of diodes used, the RF-to-DC conversion efficiency for several input powers, and finally the value of the load impedance considered. The symbol “-” indicates that the corresponding value is not mentioned in the reference.

It is possible to underline the very good performance of the proposed tri-band antenna, especially in terms of compactness and radiation efficiency. For the rectifier part, with a single identical circuit for all three bands, a fairly high RF-to-DC conversion efficiency is achieved over a relatively wide range of input power.

Therefore, the designed tri-band rectenna operating in the UHF RFID, GSM-1800, and UMTS-2100 bands is potentially a very good candidate for RF energy harvesting for very low to low input powers in the ambient environment. Specifically, the rectenna operates at resonance frequencies of 0.85, 1.8, and 2.1 GHz with a bandwidth greater than 80 MHz and presents a good RF-DC conversion performance both when the input power varies as well as the load impedance. This energy recovery device therefore offers a rather versatile behavior and is able to provide a source of energy to a component used in various environments.

**Table 4:** Comparative characteristics of the proposed tri-band antenna against related works.

Ref.	Substrate	Dimensions (mm^2^)	RF Bands (GHz)	Gain (dB)	Efficiency (%)
[9]	FR4	100 × 120	0.9, 1.8, 2.1, 2.4	4.3 - -	92 - -
[18]	FR4	80.29 × 103.41	0.86 1800 2100	1.14 2.62 4.3	- - -
[27]	Rogers 3003	200 × 175	0.9 1.8 2.1	8.15, 7.15, 8.15	- - -
[28]	FR4	60 × 60	0.9, 1.8, 2.5, 3.5, 5.5	1 2.8 4	71 - 85
Proposed antenna	FR4	78 × 50	0.85 1.8 2.1	0.1 0.8 5.6	98.5 83.3 82.4

**Table 5:** Comparative characteristics of the proposed tri-band rectifying circuit against related works.

Ref.	RF Band (GHz)	Port Number	Diode Rectifier Circuit	Diode	RF-DC Conversion Efficiency % Input Power (dBm)				Load (KΩ)
					−20	−15	−10	0	
[9]	0.9	-	Double voltage	HSMS-285C	-	-	-	60	5
	1.8				-	-	-	-	
	2.1				-	-	-	-	
	2.4				-	-	-	-	
[14]	0.9		Single serial voltage	SMS7630-079	27.3	35	42	-	5
	1.8				20	26	32	-	
	2.1				14	20	25	-	
[17]	0.85	2	Double voltage	HSMS 285B and 2 SMS 7630	48	-	-	-	1.5
	1.81, 2.18				-	-	-	-	
	2.4				-	-	-	-	
[18]	0.86	-	Double voltage	HSMS285C	-	-	20	45	14
	1.8				-	-	13	-	
	2.1				-	-	13	-	
[21]	0.9	3	Double voltage	HSMS 2852	-	22	33.7	52	3.8
	1.8				-	13	22	50	
	2.45				-	9	20	46.5	
[22]	0.9	1	Single serial voltage	HSMS 2850	-	30	39	-	3.3
	1.8				-	-	-	-	
	2.1				19.6	-	-	-	
[23]	0.9	3	Double voltage		15	-	-	60	11
	1.8				-	-	-	65	
	2.1				-	-	-	65	
	2.45				-	-	-	84	
[27]	0.9	2	Single serial	SMS 7630-079	35	35	41	-	5
	1.8				25	25	30	-	
	2.1				18	18	25	-	
[28]	0.9	3	Double voltage	HSMS-2820	-	-	2	28	
	1.8				-	-	1	9	
	2.5				-	-	2	25	
	3.5				-	-	-	-	
	5.5				-	-	-	-	
Proposed rectifying circuit	0.9	1	Single Serial diode	HSMS 285B	15	18	26	30	1.8
	1.8				21	26	33	35	
	2.1				3	8	11	16	

## 5. Conclusions

In this paper, a novel tri-band compact rectenna is proposed. The study details the design steps and simulation results obtained through to fabrication and experimental characterization.

Specifically, the antenna is a simple, double-face, printed antenna that is easy to manufacture at low cost. Regarding compactness, the size of the radiating element is only 78 × 50 mm^2^. The antenna operates at resonance frequencies of 0.85, 1.8, and 2.1 GHz with a bandwidth greater than 80 MHz. Moreover, the maximum realized gain of the antenna is 5.6 dB obtained at the frequency of 2.1 GHz, while the gains are equal to 0.1 dB and 0.8 dB for the frequency bands of 0.9 GHz and 1.8 GHz, but with a quasi-omnidirectional diagram pattern. The radiation efficiency is above 80% for all frequencies and reaches more than 98% at the 0.9 GHz frequency.

The RF-DC rectification circuit is based on a single rectifier diode structure and is compatible for the three frequency bands considered. Made of distributed elements, it is also simple to manufacture and low cost. In terms of performance, a RF-to-DC conversion efficiency of more than 30% is obtained for the frequencies 0.9 GHz and 1.8 GHz, with moreover a “plateau” effect on the input power range varying from −10 dBm to 5 dBm for a load impedance equal to 1.8 kΩ. Under these conditions, for the 2.1 GHz frequency, the efficiency increases almost linearly to reach a maximum equal to 20%. Moreover, the efficiency for the two low frequencies remains relatively high even at low input powers, a very interesting result for this type of application. Finally, for all three frequency bands, the converter’s performance also remains relatively robust to the impedance variations of the considered load.

The proposed tri-band rectenna has good overall performance and is compatible with three commonly used radio frequency bands, UHF RFID, GSM-1800, and UMTS-2100. Consequently, the proposed solution seems very promising to power all types of low power sensors by exploiting the ambient RF waves of three radio standards for indoor or outdoor applications. In future work, it would be interesting to test it in real operating conditions.

## Figures and Tables

**Table 1:** Comparison of antenna parameters between step 5 and step 6.

Step	Frequency (GHz)	S11 (dB)	Bandwidth (MHz)	Radiation Efficiency (%)
Step 5	0.94	−33.79	139	97.85
	1.81	−21.8	60	83.44
	2.08	−23.70	78	81.27
Step 6	0.93	−39.9	169	98.49
	1.83	−25.43	80	83.26
	2.11	−36	90	82.43

**Table 2:** Rectifier microstrip line dimensions.

Parameter	Value (mm)	Parameter	Value (mm)
W1	5.5	Wb	0.6
W2	4.5	W	1
W3	0.6	L1, Lb	2
W4, W6, W9, W31	1	L2	10
W5, W11, W19, W20, W21, W25, W26, W27	2	L3, L6	3
W7	4.2	L4	6
W8, W16	5	L5	1.5
W10	0.7	L7	12
W12, W29	8	L8, La	8
W13, W14	3	L9, L11	4
W15	1.5	L10	11.5
W17	2.5	L12	13.9
W18	9	A1	90
W22, W23, W28	4	A2	50
W24, Wa	0.8	A3	40
W30	6	r	2

**Table 3:** Detail of Antenna dimensions.

Parameter	Value (mm)	Parameter	Value (mm)	Parameter	Value (mm)
Ws	50	Wg1	9	W1	21
Ls	78	Wg2	10	W2	11
Wp	42	S1	2.5	R1	5
Lp	37.2	S2	5	R2	3
Wf	3	S3	2	R3	2,5
Lf	47.5	d	4	R4	1.5

## Data Availability

Not applicable.
